# Telitacicept Following Plasma Exchange in the Treatment of Subjects With Recurrent NMOSD: Study Protocol for a Single-Center, Single-Arm, Open-Label Study

**DOI:** 10.3389/fneur.2021.596791

**Published:** 2021-03-18

**Authors:** Jie Ding, Yu Cai, Ye Deng, Xianguo Jiang, Meichun Gao, Yan Lin, Nan Zhao, Ze Wang, Haojun Yu, Wenwen Lv, Ying Zhang, Yong Hao, Yangtai Guan

**Affiliations:** ^1^Department of Neurology, Renji Hospital, School of Medicine, Shanghai Jiaotong University, Shanghai, China; ^2^Clinical Research Center, School of Medicine, Shanghai Jiaotong University, Shanghai, China

**Keywords:** neuromyelitis optica spectrum disorders, telitacicept, plasma exchange, clinical study, safety, effectiveness, study protocol

## Abstract

**Background:** Neuromyelitis optica spectrum disorder (NMOSD) is an autoimmune demyelinating disease that recurrently relapses and leads to severe disability. The available choices for disease prevention are few or intolerable. Previous studies suggested that telitacicept may provide a promising therapeutic strategy for autoimmune diseases involving B cells. Therefore, this study aims to assess the effectiveness and safety of telitacicept for recurrent NMOSD.

**Methods:** We will perform a single-arm, single-center, open-label, specialist study with a total enrollment of eight participants. The treatment regimen includes plasma exchange three times and subcutaneous injection of telitacicept for 46 cycles, with a total period of 48 weeks. The primary endpoint is the time to first recurrence after enrollment. Secondary endpoints are Expanded Disability Status Scale (EDSS) score, Opticospinal Impairment Scale (OSIS) score, Hauser Ambulation Index, number of lesions on MRI, and changes in visual evoked potential (VEP), optical coherence tomography (OCT) and immunologic status. All adverse events after medication will be documented and investigated.

**Discussion:** This study will explore the safety and effectiveness of telitacicept following plasma exchange regarding the time to recurrence in neuromyelitis optica spectrum disorder (NMOSD) for the first time.

**Clinical Trial Registration:** Chictr.org.cn, identifier ChiCTR1800019427

## Introduction

Neuromyelitis optica spectrum disorder (NMOSD) is a primary B-cell mediated central nervous system autoimmune disease with an incidence of 0.5–10 per 100,000 people (1), less than the number of 60 per 100,000 people in areas with high incidence of Multiple Sclerosis(MS). However, as the diagnostic criteria of NMOSD was firstly revised in 2015 (2), there may be some missed diagnoses in current incidence of NMOSD. Current study also showed that NMOSD is more prevalent than MS in Asian population. In addition, NMOSD commonly occurs in young and middle-aged patients. It may relapse recurrently and lead to severe disability (3, 4). Therefore, it is of great importance to reduce the relapse rate to ameliorate severe neurological disability (4, 5).

Currently, infusions of high-dose intravenous methylprednisolone (IVMP) and plasma exchange (PLEX) (6) are commonly used in acute phase treatment. Drugs such as azathioprine, rituximab and mycophenolate mofetil are available immunotherapies used for patients with NMOSD to prevent relapse (5, 7, 8). However, recurrent relapse is still common in patients with NMOSD as lack of specific therapeutic target. In addition, some patients may not tolerate certain treatments because of their side effects, such as avascular necrosis of femoral head and hepatic impairment (5). Some new drugs are underway for NMOSD such as CD19 targeting antibody inebilizumab and IL-6 receptor antibody satralizumab (9). However, they are still inaccessible especially in China. Thus, it remains urgent to develop a new therapy targeting the pathogenesis of NMOSD with better effectiveness and safety, so as to remit rapidly and reduce recurrence.

NMOSD is a primary B-cell and antibody-mediated autoimmune disease (10). The release of autoantibodies from B cells indicates that there may be a specific step in B-cell receptor signaling that can be targeted to inhibit disease occurrence. B-lymphocyte stimulator (BLyS) and proliferation-inducing ligand (APRIL) both belong to the tumor necrosis factor (TNF) ligand superfamily. Accumulating evidence suggests the interplay between BLyS and APRIL plays a role in regulating B-cell maturation, function and survival (11–13). Overexpressed BLyS and APRIL are found in immune disorders such as rheumatoid arthritis (RA), systemic lupus erythematosus (SLE) and NMOSD (14–17). They are also correlated with disease activity and severity (18). Therefore, suppressing both BLyS and APRIL is also highly likely to be effective in treating NMOSD.

The regulatory role of BLyS and APRIL involves both B-cell maturation antigen (BCMA) receptors and transmembrane activator and calcium modulator and cyclophilin-ligand interactor (TACI) (19, 20). Telitacicept (previously known as TACI-Ig or Atacicept) is a recombinant fusion protein of both the ligand-binding domain of the TACI receptor and the Fc component of human IgG. Telitacicept is designed to inhibit the effects of B-cell BLyS and APRIL and may provide therapeutic potential for NMOSD (21, 22).

Studies have shown the safety and efficacy of telitacicept in B-cell-mediated diseases such as SLE and RA (23–25). Studies have also demonstrated that agents blocking BLyS and APRIL could improve clinical manifestations in a rodent model of experimental autoimmune encephalomyelitis (26). Nevertheless, telitacicept may increase clinical disease activity in patients with multiple sclerosis, a primary T-cell mediated central nervous system autoimmune disease. (27) It may also increase conversion to clinically definite multiple sclerosis in patients with unilateral symptomatic Optic neuritis as a clinically isolated syndrome (CIS) (28). However, the effectiveness and safety of telitacicept in aquaporin-4 antibody associated NMOSD patients has rarely been reported. Thus, the purpose of this study is to initially observe the effectiveness and safety of telitacicept following plasma exchange in NMOSD patients with recurrent onset.

## Methods and Analysis

### Study Design

**Figure 1:**
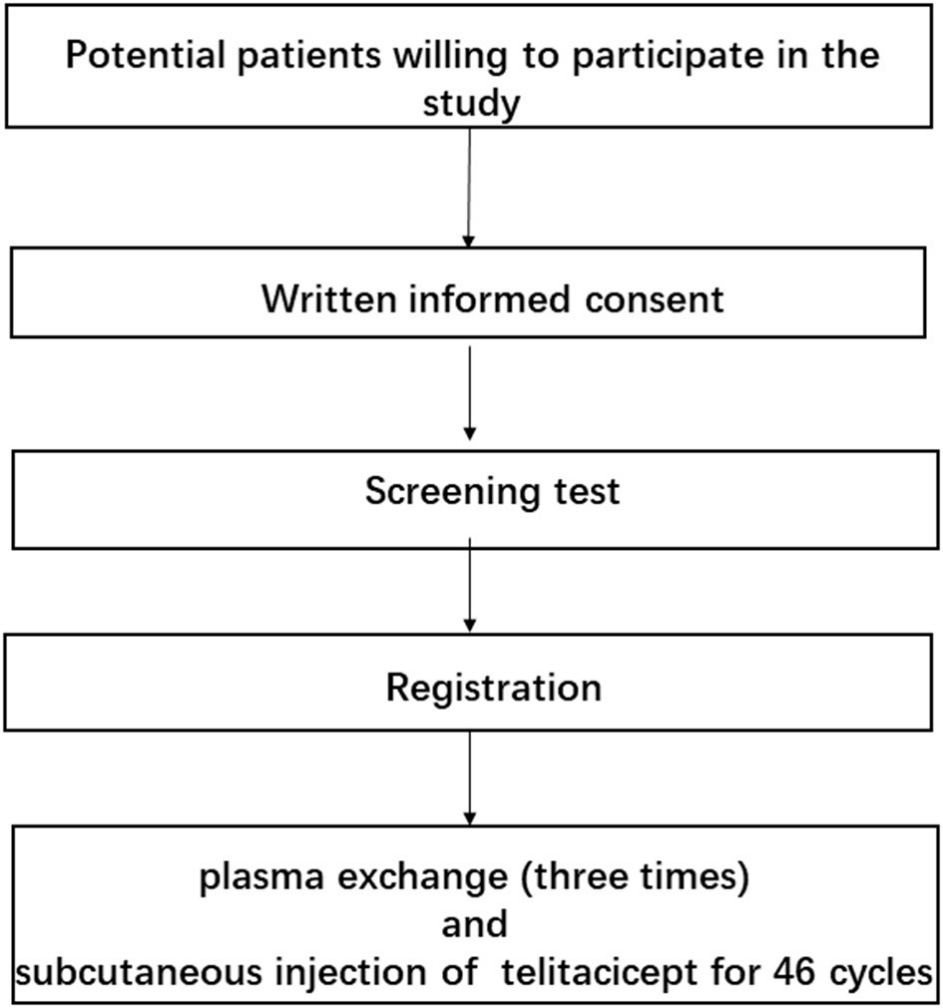
Summary of the study.

Our study is a single-center, single-arm, open-label clinical study and abide by the SPIRIT reporting guidelines (29). The purpose of this study is to assess any reduction in the incidence of recurrence in patients with recurrent NMOSD by the administration of telitacicept after plasma exchange, and safety was also analyzed. All participating individuals will receive plasma exchange three times and then undergo subcutaneous injection of telitacicept for 46 cycles, with a total period of 48 weeks (Figure 1). The study is planned to enroll eight participants.

### Sample Size

Considering the small and special nature of the study, the sample size will not be analyzed.

### Study Participants

The potential participants of this study are patients with AQP4-IgG-seropositive NMOSD in the acute phase, with ≥2 recurrences occurring in the past 12 months prior to screening (including the acute phase recurrence). Written informed consent is required for all potential participants recruited prior to screening in Renji Hospital affiliated to Shanghai Jiao Tong University, School of Medicine. After rigorous screening, the patients enrolled in this study are required to meet all the inclusion criteria without satisfying any of the exclusion criteria (Box 1).

Box 1 **Inclusion and exclusion criteria for patients.**

**Inclusion criteria**

1. Meet the diagnostic criteria for NMOSD in the 2015 international consensus; AQP4-IgG-seropositive.

2. Age 18–75 years.

3. NMOSD in the acute phase; that is, new onset of neurological symptoms or aggravation of existing symptoms within 30 days before screening, lasting for at least 24 hours without accompanying fever.

4. NMOSD recurrence (including acute onset during screening): ≥2 recurrences occurring 12 months before screening.

5. Effective contraception during the study. 6. Provide written informed consent.

**Exclusion criteria**

1. Progressively deteriorating neurological disorders unrelated to NMOSD.

2. Active hepatitis or severe liver dysfunction (over 2 times the normal value of liver function examination); HBsAg-positive patients were excluded. Patients with only HBc-Ab positivity are needed for the HBV-DNA quantitative test and are not excluded if negative.

3. Severe renal insufficiency (including acute kidney injury and chronic kidney disease, or serum creatinine clearance <60 mL/min calculated by the Cockcroft-Gault equation).

4. Current pregnancy, breastfeeding or plans to become pregnant in the past 48 weeks.

5. Participation in any clinical trial drug within 28 days before enrollment or 5 times the half-life period of the experimental drug (for a shorter time).

6. History of splenectomy.

7. Allergic reaction: history of allergies to contrast agents or human-derived biological products for parenteral administration.

8. Severe psychiatric symptoms and the inability to cooperate.

9. Unable to undergo magnetic resonance imaging.

10. Medication history of rituximab or mitoxantrone 3 months prior to enrollment.

11. Considered unsuitable by the investigators.

### Schedule of the Study

Participants providing written informed consent will undergo screening tests, and the investigators will review the criteria of patient inclusion and exclusion. If participating individuals meet all the inclusion criteria without satisfying any of the exclusion criteria, they will be documented as registered in our study. After written informed consent is obtained, the study will consist of a screening period with a maximum of 2 weeks and a period of treatment for 48 weeks. The patients will be treated with plasma exchange during the first 2 weeks of the treatment period. Afterward, telitacicept will be given weekly for 46 weeks (Table 1). After registration, the participants will not receive any treatment with glucocorticoids or immunosuppressive agents until the recurrence of NMOSD.

**Table 1:** Schedule of enrollment, interventions and assessments.

	**Study period**							
	**Preobservation**		**Treatment period**					
Visit^a^	1	2 (baseline)	3	4	5	6	7	Recurrence^j^
Timepoint (week)	−2	0	2	4	12	24	48	/
Informed consent	X							
Collection of medical history^b^	X							
Vital signs	X	X	X	X	X	X	X	X
Physical examination	X	X	X	X	X	X	X	X
Plasma exchange		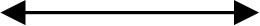						
Telitacicept	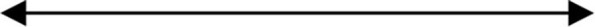							
Virology examination^c^	X				X	X	X	
Blood and urinary tests^d^	X	X*		X	X	X	X	X
Titers of AQP4-IgG^e^	X					X	X	
MRI^f^	X						X	X
Chest X-ray/chest CT^g^	X						X	X
Other tests^h^	X	X*		X	X	X	X	X
Scale score^i^	X	X*		X	X	X	X	X
Adverse events	X	X	X	X	X	X	X	X
Concomitant drugs	X	X	X	X	X	X	X	X

a *A visit once a week is needed 14 days after baseline for drug administration and to record changes in the disease, adverse events, and combination of drugs*.

b *The collected medical history included age, sex, height, body weight, nation, profession, smoking history, female reproductive history, history, date of diagnosis, medications, and history of drug allergies, complications and concomitant drugs*.

c *If the HBV-DNA quantitative test is negative for the patient, it needs to be performed at the 12th week, 24th week and 48th week during the study. If HBV-DNA is positive during the study, the patient needs to be withdrawn*.

d *Blood and urine tests included complete blood count, blood biochemistry, pregnancy test for females, titers of IgA, IgG, IgM, complement 3, complement 4, lymphocyte subsets and urinary test*.

e *AQP4-IgG should be tested including titers by the method of cell-based transfection immunofluorescence assay(CBA)*.

f *MRI examination can be performed within ±7 days*.

g *Chest radiographs within 12 weeks of the baseline visit are acceptable. Either chest X-ray or chest CT is acceptable*.

h *Other tests include ECG, visual evoked potential and optical coherence tomography*.

i *The scale score includes the EDSS score, OSIS score and Hauser Ambulation Index*.

j *Examinations need to be performed if the patient is not examined within 7 days prior to withdrawal from the study*.

* *The tests may not be repeated within 7 days*.

### Intervention

When participants are enrolled, they will be given plasma exchange three times, lasting no more than 14 days. Afterward, they will receive telitacicept treatment weekly for 46 weeks. The time lapse from onset of symptoms to first administration of telitacicept is within 17 to 58 days. The interval is at least 48 hours. A dose of 240 mg will be subcutaneously injected once. The injection site can be the thigh, abdomen or upper arm. At least one clinician needs to observe the patients for 30 min, mainly observing the patient's consciousness and vital signs.

#### Telitacicept Formulations

Telitacicept is a freeze-dried powder injection. The specification is 80 mg for one piece. It needs to be stored and transported at 2°C~8°C. After rehydration, the drug should not be left at room temperature for more than 30 min. Every 80 mg of drug will be dissolved in 1 mL of injection water into an isotonic solution.

#### Withdrawal and Reduction of the Drug

The patients will be withdrawn from the study if relapse occurs. Except for special situations, the dosage, method of administration and time interval are not allowed to be changed during the study. However, during or after the 12-week visit, the investigator will evaluate the safety of the therapy in the patient. If the investigator considers that the dose needs to be reduced to ensure safety, then the dose can be adjusted. Supposing that a dose started at 240 mg, it can be adjusted to 160 mg for the first time and adjusted again to 80 mg. The minimum dose is 80 mg.

The drug should be suspended if any of the following conditions appear: (1) cell count of leukocytes <2.0 × 10^9^/L; (2) cell count of neutrophils <1.0 × 10^9^/L; (3) cell count of lymphocytes <0.5 × 10^9^/L; (4) count of platelets <75 × 10^9^/L; (5) aspartate aminotransferase (AST) or alanine aminotransferase (ALT) > 3×upper limit of normal (ULN); or (6) hemoglobin <80 g/L. With the approval of the investigator, drug use can be continued if participants meet the following criteria: (1) cell count of leukocytes ≥2.5 × 10^9^/L; (2) cell count of neutrophils >2.0 × 10^9^/L or the count at baseline; (3) cell count of lymphocytes ≥ 0.75 × 10^9^/L; (4) count of platelets ≥100 × 10^9^/L or the count at baseline; (5) AST or ALT < 2×ULN; and (6) hemoglobin ≥100 g/L or the count at baseline. If the conditions listed above occur again after restarting the drug administration, the patient should permanently discontinue the drug. In addition, if any of the following conditions occur during the study, the patient should also withdraw from the study: (1) AST or ALT > 8×ULN; (2) AST or ALT > 5×ULN and the duration of drug suspension exceeds 2 weeks; (3) AST or ALT > 3×ULN and serum total bilirubin concentration>2×ULN; (4) AST or ALT >3×ULN along with fever, rash, nausea, vomiting, fatigue, right upper abdomen pain, or eosinophilia; (5) cell count of leukocytes <1.0×109/L; (6) cell count of neutrophils <0.5×109/L; (7) cell count of lymphocytes <0.2×109/L; or (8) hemoglobin <65 g/L.

#### Adherence

At each visit and drug administration, compliance instructions will be provided and confirmed by the investigators.

### Concomitant Drugs and Therapy

Concomitant drugs or therapies will not be applied during either the screening or treatment period. The oral or intravenous administration of glucocorticoids is not allowed; however, intranasal, intraocular, topical or inhaled glucocorticoids will be allowed. Immunosuppressants are also not allowed, such as tripterygium glycosides, cyclosporine, methotrexate, mitoxantrone, cyclophosphamide, azathioprine, tacrolimus, leflunomide, mycophenolate mofetil and teriflunomide. The following concomitant drugs and therapies are prohibited: biological immunosuppressive agents such as rituximab, hematopoietic stem cell transplantation, lymphatic irradiation and immunoglobulin injection.

Mecobalamin and vitamin B1 are allowed to be used during the study. In the case of other diseases during the study, drugs that do not affect the efficacy of the telitacicept (such as antibiotics) can be used. However, the reasons for the use of drugs and method of application should be recorded in detail.

### Outcomes

#### Primary Outcome

The primary outcome is time to first recurrence after enrollment. The definition of recurrence needs to meet the following criteria: (1) new nervous system abnormalities or worsening of existing symptoms; (2) symptoms lasting for at least 24 h; (3) over 30 days after the last relapse; and (4) no associated fever, temperature <37.5°C, or without known infection.

#### Secondary Outcomes

Secondary outcomes are listed below:

1. EDSS score
2. OSIS score
3. Hauser Ambulation Index
4. Number of lesions in MRI
5. Changes in visual evoked potential (VEP)
6. Changes in optical coherence tomography (OCT)
7. Changes in immunologic status
8. All adverse events after medication.

The EDSS score, OSIS score, Hauser Ambulation Index are evaluated by two professional physicians. Number of lesions in MRI include number of high signal lesions on T2 weighted imaging (T2WI), low signal lesions on T1 weighted imaging (T1WI) and lesions on gadolinium-enhanced T1WI, which are obtained in MRI of the optic nerve, brain and spinal cord. MRI scans are analyzed independently by radiologists at the Department of Radiology at Renji Hospital affiliated with Shanghai Jiaotong University School of Medicine. The latency and amplitude of evoked potential P100 were recorded by VEP. Optical coherence tomography (OCT) is used to measure the thickness of the retinal nerve fiber layer (RNFL), which is obtained with the “Fast RNFL Thickness” protocol. The titers of anti-AQP4 antibody are tested by the method of cell-based transfection immunofluorescence assay (CBA). Other immune tests are titers of IgA, IgG, IgM, complement 3, complement 4 in serum and lymphocyte subsets in the peripheral blood.

Adverse events will be graded using the Common Terminology Criteria for Adverse Events (CTCAE V.4.0). Common adverse events are local reactions at the injection site, including mild to moderate erythema and itchy skin rashes. Other potential adverse events include upper respiratory tract infection, bruises and itching at the injection site, soreness and redness at the injection site, urinary tract infection, herpes zoster, mumps, periodontitis, abnormality of liver function, hypothyroidism, fatigue, headache, sore throat, nasopharyngitis, diarrhea, nausea, vomiting, flu-like symptoms, cough, oral ulcer, nasal congestion, palpebral edema, right upper abdominal pain, joint pain, back pain, and perianal abscess.

### Data Analysis

Patients who are enrolled in this study and receive the test drugs at least once, with at least one post-medication evaluation, will be included in the full analysis set (FAS). The per protocol set (PPS) is described as participating individuals in the FAS who comply with the protocol, including the therapeutic regimen and the availability of the primary outcome with no major violation of the protocol. The primary outcome will be analyzed in the PPS. Participants who are administered the test drugs at least once and have recorded security evaluations will be included in the safety set (SS). Safety will be analyzed in the SS.

#### Primary Outcome

The quartile of time to recurrence, 95% CI and censoring rate will be calculated.

#### Secondary Outcomes

The secondary outcomes will be analyzed in the form of simple descriptive statistics, such as the mean and associated 95% CIs, SDs, median, minimum, and maximum. Paired *t* test will be used for statistical analyses of the difference in means before and after administration.

#### Safety Analysis

All adverse events will be analyzed in the SS. The Medical Dictionary for Regulatory Activities (MedDRA) will be used to code the preferred terms (PT) for adverse events and system organ class (SOC). All adverse events will be listed in detail, and the incidence of different adverse events will be calculated. All statistical analyses will be performed using JMP software (SAS V.9.3), with significance set at a two-tailed *p*-value.

### Data Management

All data for every participant will be recorded by investigators in case report forms (CRFs). CRFs will be made according to the plan at screening, at baseline, at 4 weeks, 12 weeks, and 24 weeks after baseline, at the end of treatment and if relapse occurs. The researcher will review and sign the CRF when complete. The investigator needs to re-sign the form if the data are modified.

### Data and Safety Monitoring

Materials such as the research records, informed consent forms and data of all patients will be reviewed every 6 months to ensure the accuracy and integrity of the data.

### Patient and Public Involvement

Patients and/or the public were not involved in the design, or conduct, or reporting, or dissemination plans of this research.

### Confidentiality

All information collected in our study will be stored securely in locked file cabinets at the study site. All information of every participating individual will be identified by a coded number without names or other personal identifiers to maintain participant confidentiality. A password will be applied to protect the database.

## Ethics and Dissemination

Our study will be performed according to the principles of the Declaration of Helsinki and has been approved by the ethics committee of Renji Hospital affiliated with Shanghai Jiaotong University School of Medicine. Investigators will explain the study by information sheets. Patients who are willing to participate will have enough time to ask questions. All questions will be explained in detail before obtaining written informed consent. All participants will be provided consent forms. Compensation will be given to those who suffer harm from trial participation.

The results of this study will be reported in academic conferences and peer-reviewed journals. The paper will be reviewed and approved by all authors before publication.

## Discussion

Recently, an increasing number of studies have focused on treatments involving biological agents in patients with NMOSD (8). Few studies have been conducted on the treatment of telitacicept in patients with NMOSD. Compared with available immunosuppressive drugs such as azathioprine and mycophenolate mofetil, telitacicept shows advantages in targeting a very specific molecule. Therefore, some off-target adverse effects may be avoided, and the treatment may be more accurate.

The results of this study may be useful for considering the safety of new biological agents in patients with NMOSD. Studies in healthy volunteers have demonstrated the safety and tolerability of telitacicept in a healthy population (30, 31). Randomized trials have also shown the safety and tolerability of telitacicept in autoimmune diseases such as SLE and RA (24, 32–34).

The study will also provide preliminary data on the efficacy of telitacicept in patients with NMOSD. Since NMOSD is a primary B cell- and antibody-mediated autoimmune disease, AQP4-IgG plays an important role in the disease (35). In our study, patients in the acute phase with AQP4-IgG seropositivity will be included. When disease occurs, plasma exchange will be first applied to filter out AQP4-IgG. Afterward, telitacicept treatment will be used to inhibit the activity of BLyS and APRIL, which may interfere with B cell function and autoantibody production. The combination of plasma exchange and telitacicept may provide better effects.

In addition, we will provide a detailed description to demonstrate the efficacy of telitacicept, including the EDSS score, OSIS score, Hauser Ambulation Index, number of lesions in MRI, and changes in OCT, VEP and immunologic status. We expect that the review of this protocol may be useful for other researchers to promote future related clinical trials.

Nevertheless, the sampling strategy is a limitation and potential source of bias in this study. We will only be able to preliminarily demonstrate the probable safety and efficacy of telitacicept, as this study is a single-center, single-arm, open-label study. Large-sample, prospective, multicenter, randomized controlled studies need to be performed in the future. In addition, the assessment of safety and tolerability will only be based on a 1-year follow-up visit. A longer follow-up period may be needed.

In this study, we expect that telitacicept may be safe and effective in patients with recurrent NMOSD; however, large sample, prospective, multicenter, randomized controlled studies are needed for further validation. Overall, as there are few studies on telitacicept treatment in patients with NMOSD, this study will be useful in presenting preliminary data for future study designs.

## Strengths and Limitations of This Study

▸ This study will explore the safety and effectiveness of telitacicept following plasma exchange regarding the time to recurrence in neuromyelitis optica spectrum disorder (NMOSD) for the first time.
▸ This study is well-designed for the selection of NMOSD patients with a high recurrence risk.
▸ A major limitation of this study is the small sample size, which weakens the power of evidence for the effectiveness of telitacicept. As the current low incidence of NMOSD, a small and special study is firstly conducted.
▸ Because eligible NMOSD patients are relatively rare, especially those with a recurrent acute phase within 30 days and a second recurrence within 12 months, this study does not include a control group, which may produce bias.

## Ethics Statement

The studies involving human participants were reviewed and approved by the ethics committee of Renji Hospital affiliated with Shanghai Jiaotong University School of Medicine (No 2018-088). The patients/participants provided their written informed consent to participate in this study.

## Author Contributions

JD wrote the initial draft of the paper. Y-TG, YH, and YC revised the paper. Y-TG, YC, and YZ designed the study. Y-TG, YC, YD, YZ, W-WL, and YL reviewed all versions of the protocol and contributed to the start of the study. Y-TG, YH, X-GJ, and JD analyzed and interpreted data. YH, X-GJ, JD, NZ, ZW, and H-JY collected the clinical data. M-CG contributed to the drug storage and electronic CRF. All authors have read and approved the final manuscript and agreed to be responsible for all aspects of the research to ensure that issues related to the accuracy or completeness of the study are properly studied and addressed.

## Conflict of Interest

The authors declare that the research was conducted in the absence of any commercial or financial relationships that could be construed as a potential conflict of interest.
